# Identification of Major Factors Influencing ELISpot-Based Monitoring of Cellular Responses to Antigens from *Mycobacterium tuberculosis*

**DOI:** 10.1371/journal.pone.0007972

**Published:** 2009-11-24

**Authors:** Steven G. Smith, Simone A. Joosten, Virginie Verscheure, Ansar A. Pathan, Helen McShane, Tom H. M. Ottenhoff, Hazel M. Dockrell, Françoise Mascart

**Affiliations:** 1 Department of Infectious and Tropical Diseases, London School of Hygiene & Tropical Medicine, London, United Kingdom; 2 Department of Infectious Diseases, Leiden University Medical Center, Leiden, The Netherlands; 3 Laboratory of Vaccinology and Mucosal Immunity, Hôpital Erasme, Université Libre de Bruxelles, Brussels, Belgium; 4 The Jenner Institute, Oxford, United Kingdom; 5 Immunobiology Clinic, Hôpital Erasme, Université Libre de Bruxelles, Brussels, Belgium; Institut de Pharmacologie et de Biologie Structurale, France

## Abstract

A number of different interferon-γ ELISpot protocols are in use in laboratories studying antigen-specific immune responses. It is therefore unclear how results from different assays compare, and what factors most significantly influence assay outcome. One such difference is that some laboratories use a short *in vitro* stimulation period of cells before they are transferred to the ELISpot plate; this is commonly done in the case of frozen cells, in order to enhance assay sensitivity. Other differences that may be significant include antibody coating of plates, the use of media with or without serum, the serum source and the number of cells added to the wells. The aim of this paper was to identify which components of the different ELISpot protocols influenced assay sensitivity and inter-laboratory variation. Four laboratories provided protocols for quantifying numbers of interferon-γ spot forming cells in human peripheral blood mononuclear cells stimulated with *Mycobacterium tuberculosis* derived antigens. The differences in the protocols were compared directly. We found that several sources of variation in assay protocols can be eliminated, for example by avoiding serum supplementation and using AIM-V serum free medium. In addition, the number of cells added to ELISpot wells should also be standardised. Importantly, delays in peripheral blood mononuclear cell processing before stimulation had a marked effect on the number of detectable spot forming cells; processing delay thus should be minimised as well as standardised. Finally, a pre-stimulation culture period improved the sensitivity of the assay, however this effect may be both antigen and donor dependent. In conclusion, small differences in ELISpot protocols in routine use can affect the results obtained and care should be given to conditions selected for use in a given study. A pre-stimulation step may improve the sensitivity of the assay, particularly when cells have been previously frozen.

## Introduction

In the absence of any reliable surrogate markers of protection against tuberculosis (TB) the monitoring of vaccine-induced immunity using an effective assay for immune markers is considered the best selection criterion for moving a new vaccine candidate forward from Phase 1 and IIa safety and immunogenicity studies through into Phase IIb and Phase 3 efficacy testing. Markers associated with protection against disease have not yet been identified, although multiple efforts are ongoing in biomarker identification and validation [1]–[3]. The production of interferon-γ (IFN-γ), a Th1 cytokine, is frequently measured as an indicator of immune activity against TB. Although its presence does not directly imply protection against development of disease, studies have revealed it to be at least an important component of a protective immune phenotype [4]–[7]. The ELISpot assay is an effective tool to enumerate the number of cells producing IFN-γ in response to a whole series of antigens, including peptides, peptide pools, proteins and crude bacterial extracts. Tailor-made selection of antigens can be made, which for vaccine trials will include specific vaccine components as well as positive and negative controls. In addition, the ELISpot assay has proven particularly sensitive in the detection of low-level responses (i.e. memory T-cells) when compared to other assays [8], [9]. The great advantages of ELISpot are the lack of assay-specific equipment essential for assay performance, especially when considering developing countries as important and necessary trial sites for Phase II and III evaluation, its relative high-throughput performance and its potential robustness.

Although ELISpot assays will yield potentially very important data, results may be influenced by variations in the protocol or even by execution of the same protocol by different laboratory members [10]. Especially for monitoring of immune responses where longitudinal follow up of individual patients or volunteers is desirable, it is extremely important to have comparable results in all assays. Monitoring immunity by ELISpot becomes even more complicated when executed at different study sites between which data will have to be compared. Depending on the exact study set up and research questions, samples can be assayed in real-time, implicating assay variation between follow up time points of each single volunteer, or all longitudinal samples from a volunteer can be analysed in a single assay to minimize inter-assay variation and theoretically increase assay sensitivity. Both strategies have their own advantages and disadvantages, the most significant being freezing and thawing of PBMCs in the case of batch analysis. Fresh and frozen cells may need different protocols to yield optimal ELISpot results. The addition of a pre-incubation step to improve assay sensitivity for frozen materials might resolve the problem of decreased signals, but side-by-side comparisons are lacking.

In this paper we analysed multiple factors that are of potential significance for ELISpot performance and identified those that will need to be harmonized between laboratories if comparisons between immune responses are to be made. As a starting point we compared ELISpot protocols used in the authors' laboratories and identified the major differences in approach (Table 1). Where appropriate, these differences may be eradicated for better unity between studies, or indeed used to identify the best approach for a given study depending on the specific conditions. Also of note are protocol steps on which many protocols agree such as the commercial source of antibody pairs. Although the reasons for this are not necessarily scientific but may be due to successful marketing and recommendation by “word of mouth”, a strong case may be made for groups who do not use these reagents to adopt them.

**Table 1:** Major differences (*and similarities*) in ELISpot protocols submitted by 4 participant laboratories.

PROTOCOL STEP	LABORATORY A	LABORATORY B	LABORATORY C	LABORATORY D
**Pre-incubation step**	Yes. Overnight +/− antigen in polypropylene tubes	Yes. Overnight +/− antigen in 48 well tissue culture plates	No	No
**Amount of anti-IFNγ coating antibody used**	100µl at 15µg/ml	100µl at 5µg/ml	50µl at 15µg/ml	50µl at 15µg/ml
**Number of cells per ELISpot well (used for test antigens)**	2.5×10^5^ or 0.625×10^5^ for Ag mixture or 0.31×10^5^ for positive control (calculated from proportion of original culture transferred)	2.5×10^5^ (calculated from proportion of original culture transferred)	1.0×10^5^	3.0×10^5^
**Time in ELISPOT plates**	48 hours	18–24 hours	18–24 hours	18–24 hours
**Culture medium used**	RPMI 1640+10% HI-FCS	RPMI 1640+10% pooled HI- AB human serum	RPMI 1640+5% autologous plasma	RPMI 1640+10% HI-FCS
***ELISpot Plates***	*PVDF-backed (Millipore, MAIPS4510)*	*As for Laboratory A*	*As for Laboratory A*	*As for Laboratory A*
***Capture Antibody***	*Anti-IFNγ (Mabtech 1-D1K)*	*“*	*“*	*“*
***Detection Antibody***	*Biotinylated anti-IFNγ (Mabtech 7-B6-1)*	*“*	*“*	*“*
***ELISpot Plate Pre-treatment***	*70% Ethanol*	*“*	*“*	*“*

## Materials and Methods

### Study Subjects, Venepuncture and PBMC Isolation

The primary goal was to obtain blood from individuals in whom T-cell responses could reliably be measured by ELISpot. For this reason, blood samples were obtained from BCG-vaccinated healthy adult or *M. tuberculosis* latently infected volunteers from participant institutes after receipt of written consent and ethical approval. All laboratories involved have experience in measuring T-cell responses to mycobacterial antigens such as *Mtb* PPD in these subjects. Venous blood was drawn and transferred to a tube containing 10 units of preservative-free sodium heparin per millilitre of blood. PBMC were separated from peripheral blood by density gradient centrifugation and transferred into a separate tube. In some cases, autologous plasma was also collected at this stage and stored until required. Viable PBMC were enumerated by eye using light microscopy and a Neubauer haemocytometer (Weber). All counts were duplicated for accuracy and non-viable cells were identified by Trypan Blue (Sigma) exclusion.

### Short-Term Cultured ELISpot

The nature of the experiments described here were such that various protocol points were varied in order to observe the effect of different approaches. The following are the basic methods for short-term cultured and *ex vivo* ELISpot assays. Where conditions were altered, this is described in the results section and in figure legends.

One million PBMC were transferred to 5 ml polypropylene tubes in 500 µl AIM-V (Invitrogen). Antigen was added to stimulation tubes (with one tube left as a control containing medium alone). Tubes were incubated overnight at 37°C (5% CO2) in a humidified incubator. At the same time, ELISpot plates were coated with anti-IFNγ capture antibody (Mabtech Ab 1-DIK). PVDF-backed ELISpot plates (MAIPS4510, Millipore) were pre-wet with 25 µl of 70% ethanol for no more than 2 minutes then washed twice with 200 µl per well sterile PBS (pH 7.4). Fifty microlitres of capture antibody was added to each well at a concentration of 15 µg/ml in PBS and plates were incubated overnight at 4°C. The following day, plates were washed with 200 µl per well of PBS five times and blocked with 200 µl per well of RPMI 1640 (Cambrex) with 10% foetal calf serum (HyClone, Perbio) for at least 1 hour at 37°C. Plates were then washed again three times with PBS. Overnight antigen-stimulated and control cells were resuspended by pipetting and 3× 125 µl (corresponding to 2.5×10^5^ input PBMC in triplicate) were transferred to ELISpot plates in triplicate. ELISpot plates were then incubated at 37°C for a further 24–48 hours. After this time, plates were emptied by flicking and wells were washed five times with 200 µl of PBS with 0.05% Tween-20 (Sigma). Anti-IFNγ–biotin detection antibody (Mabtech 7-B6-1) was diluted to 1 µg/ml in PBS with 0.5% FCS and 50 µl added to each well. Plates were then incubated at room temperature for 2 hours. Plates were washed in PBS/Tween as previously and 50 µl per well of streptavidin-ALP (Mabtech IFNγ ELISpot kit reagent) at a 1/1000 dilution in PBS/FCS was added to each well. Plates were again incubated at room temperature for 2 hours. After a final wash with 200 µl per well of PBS/Tween ×3 and PBS alone ×3, plates were developed for up to 30 minutes with BCIP/NBT^PLUS^ (Moss Inc., Maryland) following the manufacturers instructions. Reactions were stopped by washing with tap water after which plates were allowed to dry before spot counting.

### *Ex Vivo* ELISpot Assay

Plates were pre-wet and coated and blocked using the same procedure as for the cultured ELISpot with minor alterations. After blocking, PBMC without any prior manipulation following isolation from peripheral blood were added to wells. Antigen was added to stimulation wells otherwise cells were left in medium alone. The final volume was 200 µl per well. Each condition was tested in at least duplicate. *M.tb* PPD (Staten Serum Institute) was used at a final concentration of 10 µg/ml. ELISpot assays were cultured at 37°C for between 18 and 20 hours. Following incubation, the plates were washed, probed with detection antibody and streptavidin-ALP and developed using the AP Conjugate Substrate Kit (Biorad).

### Cryopreservation of PBMC

In some experiments, PBMC were cryopreserved prior to ELISpot assays. PBMC were resuspended in RPMI 1640 with 20% FCS at 2×10^7^ cells per ml and cooled on ice for 30 minutes. An equal volume of pre-cooled RPMI 1640 with 20% FCS and 20% DMSO (Sigma) was then added dropwise to the cell suspension. Cells were then distributed into cryovials (Nunc) at 10^7^ (1ml) per tube and cryopreserved at −80°C in Mr. Frosty containers (Nalgene). Vials were then transferred to liquid nitrogen after a day and stored for 4 weeks. To thaw, vials were defrosted quickly in a 37°C water bath and the contents transferred to a 15 ml centrifuge tube (Greiner) containing 2 mls of RPMI 1640 with 50% FCS. RPMI 1640 without FCS was then added to a volume of 14 ml and tubes centrifuged at 439g for 7 minutes. Pellets were resuspended in 1 ml AIM-V medium and cells counted. Recovered PBMC were enumerated by eye as described above. Viable cell recovery was routinely between 70–90%.

## Results and Discussion

### ELISpot Plate Preparation

The protocol steps used to prepare ELISpot plates for the addition of cells and antigen were found to vary between groups, e.g. type of plate used, employment of an ethanol pre-wash, wash and coating buffers used, coating antibody concentration and blocking buffer formulation. As manufacturers develop newer plates better suited to applications such as the ELISpot assay (such as using white plastic instead of clear plastic or different membrane compositions), such products are adopted by some groups whereas others are satisfied with the performance of earlier products. Also, one laboratory may have achieved acceptable results after washing wells in culture medium whereas others have been similarly happy with phosphate buffered saline (PBS) as a washing solution. It was thought that variables such as these might affect spot quality (i.e. size and density) and so lead to differences in spot number when software settings are such that they discriminate between a large fuzzy spot and a small well defined spot. When some of these conditions were compared in side-by-side assays, they had little effect on the number of spots counted (data not shown). We concluded that, in the interests of harmonisation, it would be feasible for groups to agree on steps such as these (suggested conditions for these steps are given in example ELISpot protocols S1 and S2 presented as supplementary material).

### Sample Preparation

#### Processing delays

**Figure 1:**
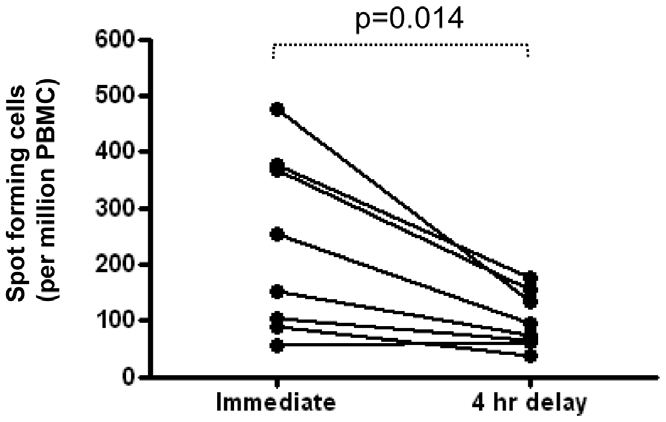
Delays in blood sample processing result in losses of ELISpot assay sensitivity. Aliquots were taken from whole blood samples of BCG-vaccinated adults (n = 8) and either processed immediately or delayed for 4 hours at room temperature before PBMC were prepared by centrifugation over Ficoll. PBMC were suspended in AIM-V serum free medium and stimulated with *M.tb* PPD (10 µg/ml) in ELISpot plates (2.5×10^5^ PBMC per well) for 18 hours prior to spot development and counting. Plates were developed according to kit manufacturers instructions (Mabtech). Results are SFC per million PBMC in antigen-stimulated samples minus that measured in medium only samples. Statistical analysis is by paired t-test.

Immunomonitoring of humans may be carried out in such a way that, in some instances, blood is taken on-site and processing can start immediately once the sample is collected, whilst in other settings, samples have to be transported some distance between the place of venepuncture and the laboratory. It has been reported by others that losses in sensitivity of short term assays (18 hrs) for T-cell function can result from delaying the processing of blood samples and the commencement of assay cultures [11], [12]. In agreement with this, we also found that the number of spot forming cells detected by ELISpot was significantly less when sample processing was delayed for four hours (whole blood, stored at room temperature) compared to immediately processed aliquots (Figure 1).

The time taken to process blood samples may be difficult to synchronize between study sites as the specific field conditions will inevitably vary. It is however apparent that this important factor will impact upon data collected using the ELISpot assay and must be considered when analyzing results. One approach would be to decide on a length of time to delay sample processing at *all* sites based on the site where the longest delay is inevitable.

### Assay Procedure

#### Media and sera

Protocol variations in steps involving the culture of PBMC with antigen in ELISpot assays were suspected to have the greatest capacity to alter the assay outcome. It was apparent from a comparison of protocols used in our laboratories and those of others that culture medium composition represented a variable in different laboratories. Examples of the use of media supplemented with foetal calf serum, human pooled AB serum and autologous sera were found as well as the use of serum-free medium alternatives [13].

**Figure 2:**
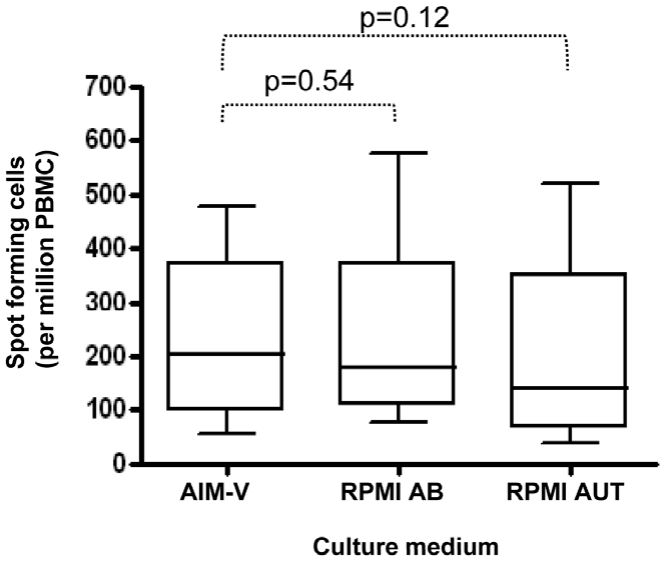
A comparison of the effects of different culture media used in ELISpot assays. Spot numbers in un-stimulated and in *M.tb* PPD stimulated *ex vivo* ELISpot assays were determined following the use of different culture media (AIM-V serum-free medium; RPMI 1640 medium supplemented with heat inactivated human pooled AB serum (RPMI AB) or RPMI 1640 medium supplemented with autologous serum (RPMI AUT). Results are SFC per million PBMC in *M.tb* PPD-stimulated samples minus that measured in medium only samples. Box and whisker plots show the median, upper and lower quartiles and range of the response (n = 8). Statistical analysis is by paired t-tests.

As the use of serum could introduce variability over time as it becomes necessary to introduce fresh serum batches, we were interested in the possibility of substituting serum-supplemented medium for a serum free alternative without the loss of assay sensitivity. We found that the measurable ELISpot response to PPD in BCG vaccinated individuals in serum free AIM-V medium was comparable to responses measured with either autologous or pooled AB human serum and that the lack of serum did not result in measured responses that were significantly any less than those measured in assays where serum was used (Figure 2). The median response to PPD in AIM-V medium was 203 spot-forming cells per million PBMC and the interquartile range 97–373. The corresponding data for un-stimulated controls was 10 (6–15). These data indicate that AIM-V is capable of detecting both high and low responses as well as ensuring a high signal to background ratio (20∶1 in this case). Thus, substitution of serum containing media with AIM-V synthetic medium would eliminate a potential source of variation. As the avoidance of serum supplementation is an easily achievable way to reduce inter-assay variation between laboratories as well as in a single laboratory over time for longitudinal studies, the adoption of a serum free medium such as AIM-V would be an effective way to ensure assay uniformity.

#### Cell numbers used

One of the most relevant considerations when deciding how many cells to add to ELISpot wells may be the expected magnitude of the response to be measured. Predicting this magnitude should help to avoid the outcome of uninformative “blacked out” wells (containing too many spots to count reliably) for strong responses or, conversely, too few spots to count when responses are weak. The nature of the immune response being tested and the recall antigen used to monitor responses will often affect this decision. If, for example, a potent vaccine regime (e.g. a prime-boost approach) is used and/or if the recall stimulus is particularly immunogenic, the number of spots detectable will be greater. In these circumstances, fewer cells may be added to ELISpot wells in order to avoid black out. The reverse is true when the vaccine or stimulus is weaker. More cells may be required to achieve enough spot-forming cells to exceed the threshold of detection in a given well.

**Figure 3:**
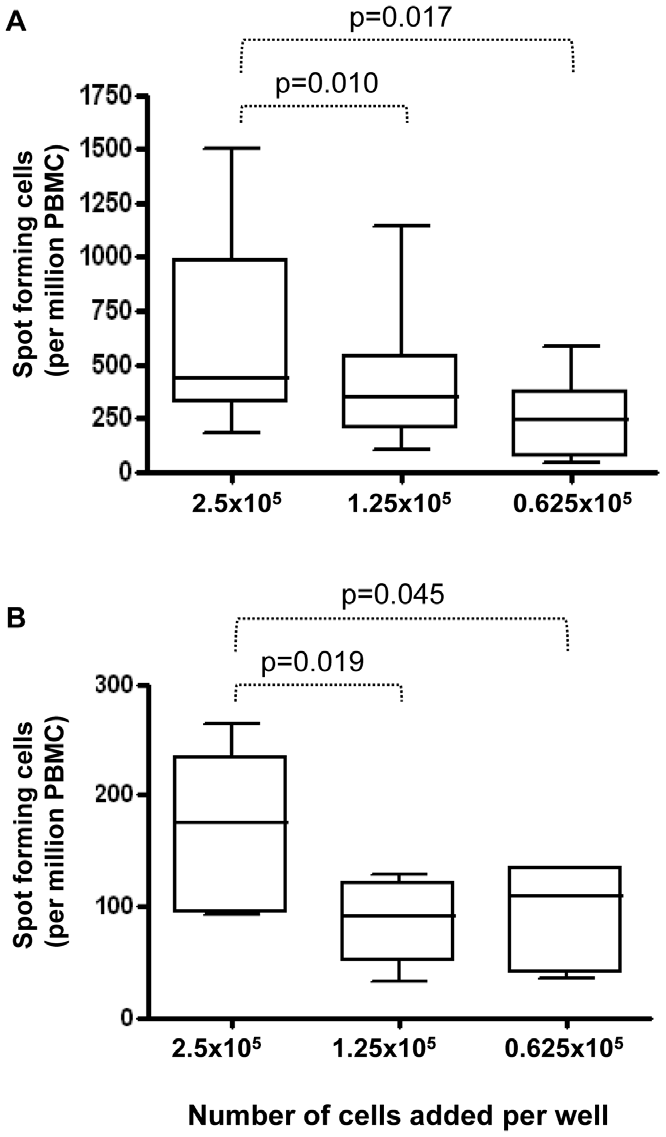
The effect of input PBMC number per well on ELISpot assay performance. Spot numbers in (A) *M.tb* PPD stimulated, *ex vivo* ELISpot assays (n = 8) or (B) HBHA-stimulated, short-term cultured ELISpot assays (n = 6) were determined following the addition of varying PBMC numbers per well. All results were obtained after the subtraction of background spots (measured in unstimulated wells). Box and whisker plots show the median, upper and lower quartiles and range of the response. Data shown are representative of similar experiments carried out in 3 of the 4 laboratories involved. Statistical analysis is by paired t-tests.

It might be assumed that for one set of assay conditions, the number of spots counted after varying the number of cells added to a well would be normalized (by calculating the number of spots per million PBMC) to give the same result. However, we found that for a given donor and stimulus, altering the number of cells in a well does not equate linearly to the same overall response when spot counts are converted to spot forming cells per million PBMC. This was demonstrated for both PPD in the standard *ex vivo* ELISpot assay (Figure 3A) and for the protein antigen HBHA in an ELISpot assay following a pre-incubation step with antigen (Figure 3B). In other words, there is not necessarily a linear relationship between the number of cells added to a well and the number of spots produced in that well. It is likely not possible to add equal cell numbers for stimulants as disparate as the positive control anti-CD3 and (protein) antigens such as HBHA [14]–[16] and expect readable, useful spot counts for both. Hence, when responses to particular stimuli such as PPD or anti-CD3 are to be measured at different sites, efforts should be made to ensure that the same numbers of cells are indeed being added to wells for a given stimulus and that these numbers relate to the expected magnitude of response and hence the number of spots per well. Precision in the counting of cells and of pipetting technique are essential considerations in order to achieve the desired number of cells per well. Repeat counts (manual or automated) that are averaged and properly calibrated pipettes are therefore vital. Given that, as yet undefined mechanisms result in measured responses that are not proportional when different input cell numbers are used, the approach outlined above should allow meaningful comparisons between responses to a given antigen. Assuming the number of stimuli to be tested is not large it may be necessary to include separate medium control wells for each cell number used as background spot counts may also be affected.

#### *Ex vivo* ELISpot assay or short-term cultured ELISpot assay

**Figure 4:**
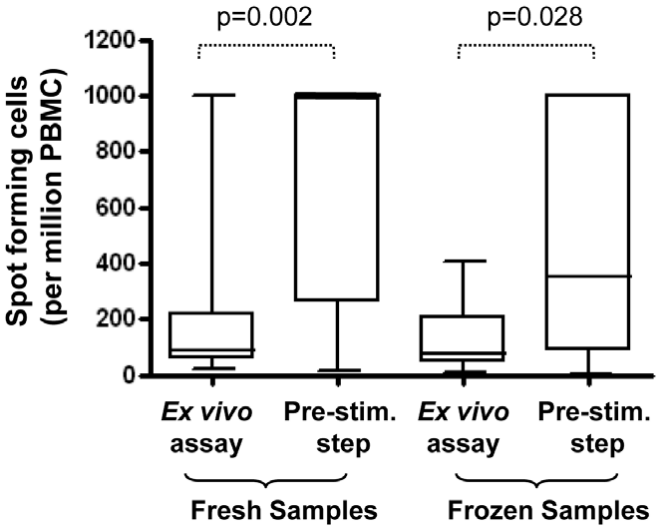
Comparison of “ex vivo” and short-term cultured “pre-stimulation” ELISpot techniques. *M.tb* PPD-stimulated responses were determined using either the *ex vivo* approach on fresh (n = 14) and cryopreserved (n = 10) or using the pre-stimulation (STC) approach on fresh (n = 12) or cryopreserved (n = 14) PBMC samples. Results are combined data from donors tested in 4 laboratories. Unreadable (blacked out) ELISpot readings were allocated a count of 1000 SFC/million PBMC. Box and whisker plots show the median, upper and lower quartiles and range of the response. Statistical analysis is by paired t-tests.

If the definition of an ELISpot assay is restricted to the period during which cells are cultured with stimulant in a well coated with capture antibody and the subsequent processing of that plate with detection antibody and colour development reagents, a major difference in approach we encountered was the inclusion of a PBMC stimulation step prior to the ELISpot assay; an approach that is termed a short-term cultured (STC) ELISpot assay. This pre-stimulation step was carried out either in multi-well tissue culture plates or in polypropylene centrifuge tubes and comprised the culture of cells with stimulant for a defined period, prior to their addition to coated ELISpot wells. The rationale for this approach is to increase the assay's sensitivity when study conditions exist in which responses may be less marked, as well as to decrease the background due to the presence of dead cells (in the case of frozen samples only). These conditions may relate to the immune response to be measured or the recall antigen used as discussed, or particularly when longitudinal studies require that samples from different time points are frozen and processed later in batches, to minimize variation in test responses that could well be caused by temporal inter-assay variability. In the current study we attempted to determine whether the STC ELISpot assay did indeed improve the sensitivity of the assay above that of a direct, “*ex vivo*” assay and whether this was indicated by greater spot counts for otherwise identically treated samples. When this comparison was carried out at the four sites, we observed a mixed outcome. When responses to *M.tb* PPD were investigated, although there was considerable variation between laboratories, as represented by the data spread within each group, the combined results from all four laboratories indicated that a pre-stimulation did increase the number of spot forming cells detected over those observed in parallel *ex vivo* assays, and that this was true for both fresh and cryopreserved samples (Figure 4). Results were less consistent when responses to all antigens tested (including PPD, protein and peptide antigens) were considered. When analysis was restricted to either protein antigens expressed in BCG (Ag85A, Ag85B) and to samples from donors known to be BCG vaccinated but not TB exposed, or to a latency protein antigen tested in latently infected subjects (HBHA), the pre-stimulation step again increased the number of spot forming cells detected (data not shown). The variation in responses observed by each laboratory may be due to the differing backgrounds of volunteer donors; e.g. age, BCG vaccine and travel history or possible exposure to TB. Despite this, it seems that for studies in which immune responses may be less marked the inclusion of a pre-stimulation step might be considered in order to improve the sensitivity of the ELISpot assay.

### Plate Reading

Even in situations where ELISpot assay conditions have been harmonized across sites as completely as possible, comparable results will depend upon the accurate enumeration of spots in each well. As the ELISpot technique has been adopted by different laboratories over time, various types of spot counting instruments and versions of the accompanying software have been obtained by each. Without a standardised approach to instrument set-up and with spot counting parameters being set by individual users in each laboratory one might expect a degree of variability when different laboratories are given the same ELISpot plate to count.

**Figure 5:**
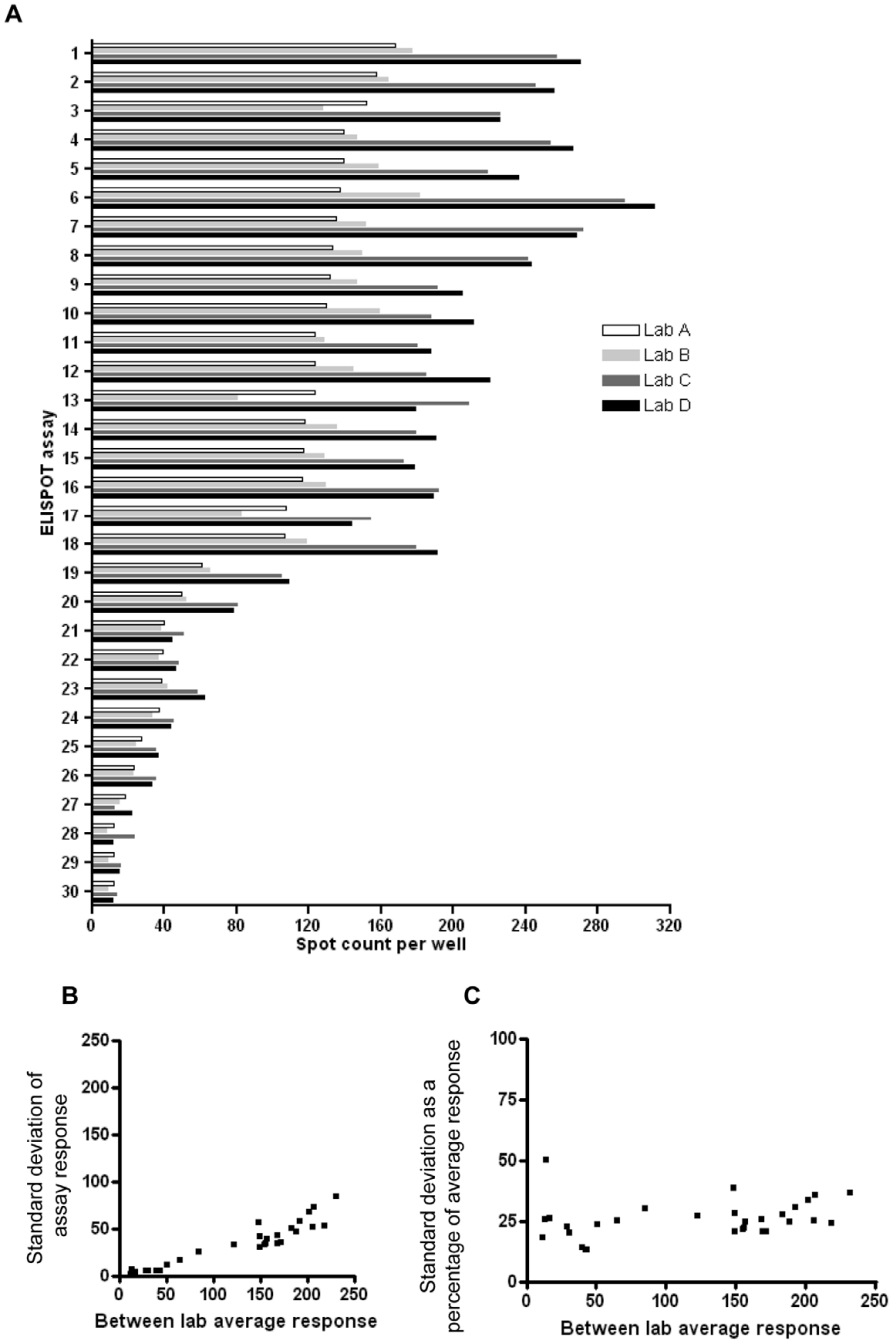
ELISpot reference plate counts obtained independently from four laboratories. Results show spot counts per well for each of 30 separate “assays” (i.e. results from donor PBMC stimulated independently with conditions varied) (A); the standard deviation of responses between laboratories as a function of the average between laboratories response (B) and the standard deviation as a proportion of the between laboratories mean response (C).

Two ELISpot plates containing 30 separate assays were circulated between the four laboratories involved in this study. The details of each of the 30 assays (10 separate conditions tested in triplicate) are described in Table 2. Each laboratory counted these according to their own settings for the counting of IFN-γ ELISpot plates using automated readers (Figure 5A). A degree of variability was indeed observed. Interestingly, 2/4 laboratories obtained similar counts that were appreciably different to those obtained by the remaining 2 laboratories, between which there was again a good degree of agreement. The degree of variability between results produced by the four laboratories (represented as the standard deviation from the mean) increased as the mean response in a particular assay increased (Figure 5B). However, Figure 5C shows that when considered as a percentage of the mean response, the standard deviation remained consistent over the range of response magnitudes (Figure 5C). Together, these two findings suggest that there is less variability between counts produced by different laboratories when responses are lower overall. The implication of this is that laboratories are more likely to agree when responses are close to the borderline between positive and negative resulting in a similar ranking of responses.

**Table 2:** Description of assays represented in Figure 5 for which each laboratory provided separate spot counts.

ASSAY CONDITIONS	ASSAY NUMBER
Human AB serum; Coating mAb – 50µl	1, 11, 24
Human AB serum; Coating mAb – 100µl	3, 15, 21
Autologous serum; Coating mAb – 50µl	2, 16, 27
Autologous serum; Coating mAb – 100µl	13, 17, 28
AIM-V serum-free medium; Coating mAb – 50µl	5, 14, 25
AIM-V serum-free medium; Coating mAb – 100µl	9, 10, 22
PPD conc. 5 µg/ml; sample handling – no delay	6, 8, 26
PPD conc. 10 µg/ml; sample handling – no delay	4, 7, 23
PPD conc. 5 µg/ml; sample handling – delayed 4 hr	18, 20, 30
PPD conc. 10 µg/ml; sample handling – delayed 4hr	12, 19, 29

Although automated counting is the most easily employed method to achieve consistency, spot counting hardware and software will often vary between laboratories. As seen here, the counting of two plates can vary between sites which have different readers and software. In the absence of matching equipment, an approach would be to circulate a batch of ELISpot plates around participating sites and allow the adjustment of machines and software settings in order that all counters read the same number of spots for those plates. This process would be facilitated by prior agreement as to the characteristics of spots that are to be counted as opposed to those that may be considered background spots and ignored. The important characteristics to consider are usually spot size, spot density, spot shape and the “fuzziness” of a spot's edges. Alternatively, all plates may be sent (blinded) to and read by one centre on a single instrument by one operator. The easiest approach may simply to ensure that all sites involved in a study have matching hardware, software and instrument settings before commencement.

### Analysis

Depending on how results are to be presented, it may be necessary to determine parameters for analysis such as a cut off point between positive and negative ELISpot assay results. The most common approach is the requirement that for a positive response to a given antigen, the average number of spots counted in the test wells is at least double that counted in the control wells (usually cells cultured in medium alone) and also that there is a difference of at least 5 spots between control and test wells. This is the criterion utilised when the ELISpot assay is employed commercially to identify latent TB and has a history of use in studies where ELISpot has been used [17]–[20].

### Conclusion

The ELISpot assay is a widely used method for detecting T-cell responses to antigens of interest, but variations in protocol can alter the results obtained. Such differences jeopardize comparisons made, for example between different vaccine candidates tested in different trials with alternative assay procedures. The recommendations presented here provide a means by which all sites participating in a trial or comparative trials of novel TB vaccines, or indeed any multi-centre study where immunological monitoring by ELISpot assay is desirable, can ensure that variations due to assay procedure are kept to a minimum. We see our findings as indicators of where differing ELISpot protocols might be altered to make them more comparable. Although the implications of some of this study's comparisons for complete assay harmonisation within the field are limited by sample sizes of 8 or less (e.g. serum usage) we believe that these results provide a useful guide as to where more comprehensive harmonisation might begin. Similarly, we acknowledge that the list of possible factors that contribute to variations in the sensitivity and specificity of the ELISpot assay is extensive and those covered here represent only a few. We believe however that the impact of those mentioned is potentially great and that these are important factors to address.

To summarise, although unlikely to markedly affect assay performance, plate preparation, washing and blocking procedures should be consistent. We recommend the use of PBS for washing, antibody dilution and blocking (when supplemented with serum) for the simple reasons that it is easiest (and cheapest) reagent on which groups can agree and it has been used successfully for these purposes in the past. In order to minimize assay variations, a serum free medium such as AIM-V is recommended for the cell culture step and for a given study and recall antigen, the number of cells added to wells should be the same at all sites. Every effort should be made to ensure that samples undergo similar treatment at all sites including the time taken from venepuncture to PBMC preparation, dilution in medium and addition to ELISpot plates. When employed, cryopreservation and thawing procedures should be consistent, agreed beforehand and harmonized on issues of freezing media, batches of foetal calf serum and dimethyl sulfoxide and use of Nalgene® Mr. Frosty containers.

The *ex vivo* ELISpot assay (see Protocol S1) is recommended for use on freshly prepared PBMC and when a strong T-cell response is predicted, e.g. in vaccine trials for prime-boost regimes or novel live vaccines. The short-term cultured ELISpot assay (see Protocol S2) is recommended for use when sample arrival is unpredictable or when weaker T-cell responses are expected, e.g. when PBMC samples have been cryopreserved and banked for batch processing.

Where possible, the same spot counting procedure, equipment and software (including settings) should be used. In addition (and especially when equipment or software differs) reference plates should be circulated between sites and equipment should be configured in such a way that all sites obtain the same readings from these plates.

ELISpot harmonisation is an important consideration before performing large scale immune-monitoring studies such as clinical trials of novel vaccines. We present here some considerations and recommendations that should facilitate the implementation of strategies for such harmonisation.

## Supporting Information

Protocol S1 Recommended protocol for carrying out the *ex vivo* ELISpot assay based on findings in paper
(0.05 MB DOC)

Protocol S2 Recommended protocol for carrying out the short-term cultured (STC) ELISpot assay based on findings in paper
(0.05 MB DOC)
