# Supplementary material for: Identification of Major Factors Influencing ELISpot-Based Monitoring of Cellular Responses to Antigens from Mycobacterium tuberculosis
Source: PLoS One. 2009 Nov 24;4(11):e7972. doi: 10.1371/journal.pone.0007972 (PMC2776358; doi:10.1371/journal.pone.0007972)
Supplement: Protocol S1 — Recommended protocol for carrying out the ex vivo ELISpot assay based on findings in paper (0.05 MB DOC) [file pone.0007972.s001.doc]

**Standard Operating Procedure**

**TBVAC – funded by the European Commission 6^th^ Framework Programme**

**SOP number SOP – TBVAC EVE 1**

**SOP title *Ex vivo* IFNγ ELISPOT**

**Date July 2009**

**Author(s) TBVAC Work Package 4**

# 1. Purpose and scope

To measure the number of peripheral blood mononuclear cells that secrete IFNγ in response to selected stimulants. This procedure will enable monitoring of cellular immune responses following participation of individuals in TB vaccine trials. It should be used when sample arrival is expected and pre-coated ELISPOT plates may be prepared in advance and when strong immune responses are expected, e.g. to very immunogenic vaccine formulations.

# 2. Responsibilities

Should only be performed by trained laboratory staff

# 3. Safety issues

All personnel performing this assay should wear disposable gloves and be vaccinated against hepatitis B. Chemicals and reagents should be handled in accordance with manufacturer’s safety recommendations.

# 4. Reagents

PVDF-backed ELISPOT plates (MAIPS4510, Millipore)

Phosphate buffered saline

Anti-IFNγ mAb (1-D1K, Mabtech)

Foetal Calf Serum

AIM-V (31035-025 - Invitrogen)

Anti-CD3 mAb (3605-1-50 - MAbtech)

Tween 20 (Sigma)

Biotinylated anti-IFNγ mAb (7-B6-1, Mabtech)

Streptavidin-ALP conjugate (Mabtech)

AP Conjugate Substrate Kit (170-6432 – Biorad)

# 5. Procedure

Day 1

- Pre-wet PVDF ELISPOT plate with 25 μl per well of 70% ethanol in distilled water for no longer than 2 minutes. Empty by flicking.
- Add 200 μl per well sterile PBS to wash and flick off. Repeat wash.
- Add 50 μl per well of coating antibody (Anti-IFNγ mAb 1-D1K) diluted to 15 μg/ml in sterile PBS (pH 7.4).
- Incubate overnight at 4˚C.

Day 2

- Flick off coating antibody and wash plate x3 with 200 μl per well of sterile PBS.
- Block plate with 50 μl per well of sterile PBS with 10% heat-inactivated foetal calf serum for 2-5 hours at 37˚C.
- Wash plate x3 with 200 μl sterile PBS.
- Add 2.5x10^5^ PBMC per well in 180μl AIM-V medium.

- Add 20 μl per well of antigen/mitogen in AIM-V medium at 10x the desired concentration or medium only (e.g. Final conc. for PPD, HBHA and all recombinant protein antigens = 10 μg/ml, for PHA = 5 μg/ml, for anti-CD3 mAb (3605-1-50 - MAbtech) = 0.1μg/ml)
- Attach the top of the ELISPOT plate with Millipore tape and incubate for 18-20 hours at 37˚C.

Day 3

- Flick off cells and wash plate x5 with 200 μl per well of PBS/0.05% Tween 20 (wash buffer).
- Add 50 μl per well of biotinylated anti-IFNγ at 1 μg/ml (1/1000 dilution in sterile PBS + 0.5% FCS).
- Incubate at room temperature for 2 hours.
- Wash plate x5 in wash buffer as above.
- Add 50 μl per well of streptavidin-ALP diluted 1/1000 in sterile PBS + 0.05% FCS.
- Incubate at room temperature for 2 hours.
- Wash plate x3 with PBS/Tween and x3 with sterile PBS.
- Add 50 μl per well of AP Conjugate Substrate Kit (Tissue Culture grade water + 1/25 AP kit diluant + 1/100 solution A +1/100 solution B).
- Incubate at room temperature in the dark for up to 30 minutes depending upon spot development.
- Wash plate thoroughly with tap water (removing plastic tray to get well bottoms) and allow drying overnight in the dark before reading.
